# EEG alpha reactivity and cholinergic system integrity in Lewy body dementia and Alzheimer’s disease

**DOI:** 10.1186/s13195-020-00613-6

**Published:** 2020-04-22

**Authors:** Julia Schumacher, Alan J. Thomas, Luis R. Peraza, Michael Firbank, Ruth Cromarty, Calum A. Hamilton, Paul C. Donaghy, John T. O’Brien, John-Paul Taylor

**Affiliations:** 1 Translational and Clinical Research Institute, Faculty of Medical Sciences, Newcastle University, Campus for Ageing and Vitality, Biomedical Research Building 3rd floor, Newcastle upon Tyne, NE4 5PL UK; 2 IXICO Plc, London, EC1A 9PN UK; 3 Department of Psychiatry, School of Medicine, University of Cambridge, Cambridge, CB2 0SP UK

**Keywords:** Resting state EEG, Structural MRI, Dementia with Lewy bodies, Parkinson’s disease dementia, Nucleus basalis of Meynert

## Abstract

**Background:**

Lewy body dementia (LBD), which includes dementia with Lewy bodies (DLB) and Parkinson’s disease dementia (PDD), is characterised by marked deficits within the cholinergic system which are more severe than in Alzheimer’s disease (AD) and are mainly caused by degeneration of the nucleus basalis of Meynert (NBM) whose widespread cholinergic projections provide the main source of cortical cholinergic innervation. EEG alpha reactivity, which refers to the reduction in alpha power over occipital electrodes upon opening the eyes, has been suggested as a potential marker of cholinergic system integrity.

**Methods:**

Eyes-open and eyes-closed resting state EEG data were recorded from 41 LBD patients (including 24 patients with DLB and 17 with PDD), 21 patients with AD, and 40 age-matched healthy controls. Alpha reactivity was calculated as the relative reduction in alpha power over occipital electrodes when opening the eyes. Structural MRI data were used to assess volumetric changes within the NBM using a probabilistic anatomical map.

**Results:**

Alpha reactivity was reduced in AD and LBD patients compared to controls with a significantly greater reduction in LBD compared to AD. Reduced alpha reactivity was associated with smaller volumes of the NBM across all groups (*ρ* = 0.42, *p*_FDR_ = 0.0001) and in the PDD group specifically (*ρ* = 0.66, *p*_FDR_ = 0.01).

**Conclusions:**

We demonstrate that LBD patients show an impairment in alpha reactivity upon opening the eyes which distinguishes this form of dementia from AD. Furthermore, our results suggest that reduced alpha reactivity might be related to a loss of cholinergic drive from the NBM, specifically in PDD.

## Background

Lewy body dementia (LBD) includes dementia with Lewy bodies (DLB) and Parkinson’s disease dementia (PDD) and is the second most common form of neurodegenerative dementia after Alzheimer’s disease (AD) [1]. LBD is characterised by Parkinsonism, fluctuating cognition, rapid eye movement sleep behaviour disorder, and visual hallucinations [2].

Both LBD and AD patients show marked cholinergic deficits which are more severe in LBD compared to AD and occur earlier in the course of the disease [3, 4]. In LBD, the cortical cholinergic deficit is mainly caused by degeneration of cholinergic nuclei within the basal forebrain [5]. The substantia innominata forms part of the basal forebrain and contains the nucleus basalis of Meynert (NBM) whose cholinergic neurons have widespread connections to the entire cortex providing the main source of cortical cholinergic innervation [6]. Several studies have identified structural abnormalities within the substantia innominata in LBD which are more pronounced than in AD [7, 8].

Cholinergic deficits are believed to be one of the major causes of cognitive dysfunction in dementia. In Parkinson’s disease, loss of cholinergic neurons in the NBM and cortical cholinergic dysfunction are related to the development of dementia [9, 10] and NBM degeneration is predictive of future cognitive decline [11]. In AD patients, NBM volume is correlated with overall cognitive performance [12].

In particular, cognitive fluctuations, which are a common symptom of both DLB and PDD, have been linked to imbalances within the cholinergic system [13]. Structural abnormalities in the substantia innominata have been shown to be related to the severity of cognitive fluctuations in DLB [7], and cholinesterase inhibitors can ameliorate this clinical symptom [14].

Previous studies have found an association between cholinergic innervation from subcortical structures and cortical neural signals as measured by EEG. In resting, eyes-closed conditions, a prominent rhythm in the alpha frequency range can be observed in the EEG which results from the synchronous firing of many cortical neurons summing to form large-amplitude signals [15]. In the healthy brain, opening of the eyes leads to a marked attenuation of alpha power due to desynchronization of neuronal activity [15, 16]. The magnitude of this reduction in alpha power from eyes closed to eyes open—termed alpha reactivity—has been suggested as a marker of cholinergic system integrity [17, 18].

Studies in AD found a decrease in alpha reactivity compared to healthy controls [19, 20]. This reduced alpha reactivity in AD has been shown to be related to the severity of cognitive impairment and was also observed in individuals with amnestic mild cognitive impairment (MCI) who are at an increased risk of developing AD, suggesting that changes in alpha reactivity might occur early in the course of the disease [20].

Franciotti et al. [21] investigated differences in alpha reactivity between AD and DLB patients using magnetencephalography (MEG). While they observed a decrease in alpha reactivity in the dementia groups compared to controls, they did not find differences between AD and DLB which might be due to the small sample size including only seven DLB patients [21]. Similarly, Bosboom et al. [22] found a reduction in alpha reactivity in PDD patients compared to controls using MEG.

However, no previous study has investigated differences in alpha reactivity between AD and LBD in a larger cohort, and how changes in alpha reactivity relate to cholinergic system integrity in these patients.

The first aim of the present study was therefore to assess alpha reactivity in a cohort of LBD patients compared to AD patients with similar levels of cognitive impairment and to healthy age-matched controls. We hypothesised that alpha reactivity would be reduced in the dementia groups with a more pronounced decrease in LBD compared to AD given the more severe cholinergic deficit in the former group [3]. The second aim was to investigate the association between changes in alpha reactivity and volumetric changes within the cholinergic system in LBD and AD. Here, we hypothesised that a reduction in alpha reactivity would be related to reductions in NBM volume in the dementia groups. Based on evidence from previous studies suggesting a link between cognitive fluctuations and disturbances within the cholinergic system [7], we also hypothesised that a reduction in alpha reactivity would be related to more severe cognitive fluctuations in LBD.

## Methods

### Participants

This study involved 102 participants over 60 years of age. Forty-one were diagnosed with probable LBD (24 DLB and 17 PDD), 21 were diagnosed with probable AD, and 40 were healthy controls of similar age with no history of psychiatric or neurological illness. Patients were recruited from the local community-dwelling population who had been referred to old-age psychiatry and neurology services. The study was approved by the local ethics committee, and written informed consent was obtained from all participants. Dementia diagnoses were performed independently by two experienced clinicians in alignment with consensus criteria for probable DLB [2], PDD [23], and AD [24]. Patients who were taking dopaminergic medication were assessed in the “ON” motor state.

All participants underwent detailed neurological and neuropsychiatric testing including the Mini-Mental State Examination (MMSE) as a measure of global cognition, the Unified Parkinson’s Disease Rating Scale part III for the assessment of Parkinsonian motor problems, and the Neuropsychiatric Inventory (NPI) hallucinations subscale which was specifically focussed on the occurrence of visual hallucinations. For the assessment of cognitive fluctuations, we used the Mayo Fluctuation Scale [25].

### EEG acquisition and pre-processing

Resting state EEG recordings were acquired from all participants using Waveguard caps (ANT Neuro, The Netherlands) comprising 128 sintered Ag/AgCl electrodes that were placed according to the 10–5 system. Participants were seated during the recording and instructed to remain awake. Electrode impedance was kept below 5 kΩ, and continuous EEG data were recorded at a sampling frequency of 1024 Hz. One hundred fifty seconds of eyes-closed and 150 s of eyes-open data were recorded from each participant. Participants were supervised by the EEG technician during the recording to monitor adherence to the protocol (i.e. eyes open vs eyes closed). The ground electrode was attached to the right clavicle, and all EEG channels were referenced to Fz during recording.

Pre-processing of eyes-closed and eyes-open EEG data was performed blinded to group membership, and the methods applied were the same as described in [26]. Briefly, data were filtered between 0.3 and 54 Hz using a second order Butterworth filter, noisy EEG segments and noisy EEG channels were deleted, and independent component analysis was used for artefact removal. The deleted channels were then replaced using spherical spline interpolation, and data were recomputed against the average reference and split into non-overlapping epochs of 2 s. For each participant, the first 45 2-s long artefact-free epochs from the eyes-closed data and the first 45 2-s long artefact-free epochs from the eyes-open data were selected for further analysis.

### Alpha reactivity analysis

EEG data from three occipital electrodes (O1, O2, and Oz) were selected for the alpha reactivity analysis (Fig. 2a) [17]. For each electrode separately, the power spectral density (PSD) was estimated using Bartlett’s method with a frequency resolution of 0.25 Hz and a Hamming window. The resulting PSD for the three electrodes was averaged across the 45 epochs and across the three electrodes for each condition separately (eyes open and eyes closed). Alpha reactivity was then calculated according to the following formula [17]:

$$ \mathrm{alpha}\ \mathrm{reactivity}=\frac{\mathrm{alpha}\ \mathrm{power}\ \mathrm{eyes}\ \mathrm{closed}-\mathrm{alpha}\ \mathrm{power}\ \mathrm{eyes}\ \mathrm{open}}{\mathrm{alpha}\ \mathrm{power}\ \mathrm{eyes}\ \mathrm{closed}} $$

where alpha power was computed as the relative power within a frequency bin around the individual alpha peak frequency ± 2 Hz. Individual alpha peak frequencies were calculated by finding the peak in the PSD in the extended alpha frequency band from 4 to 14 Hz [20] using the eyes-closed data. Individual alpha peak frequencies were used instead of the standard alpha frequency band to account for a shift of the alpha peak to slower frequencies in AD and LBD [26, 27].

### MRI acquisition and pre-processing

There were 40 healthy controls, 19 AD, 20 DLB, and 16 PDD participants from the alpha reactivity analysis with structural MRI data. MR images were acquired on a 3-T Philips Intera Achieva scanner with a magnetization prepared rapid gradient echo (MPRAGE) sequence, sagittal acquisition, echo time 4.6 ms, repetition time 8.3 ms, inversion time 1250 ms, flip angle = 8°, SENSE factor = 2, and in-plane field of view 240 × 240 mm^2^ with slice thickness 1.0 mm, yielding a voxel size of 1.0 × 1.0 × 1.0 mm^3^.

Pre-processing of MR images was performed in SPM12 (http://www.fil.ion.ucl.ac.uk/spm/). First, images were segmented into grey matter, white matter, and cerebrospinal fluid. The segmented grey matter images were then coregistered and normalised to MNI space using SPM’s DARTEL algorithm [28] and modulated. As a final step, images were smoothed with a 4-mm full width at half maximum Gaussian kernel.

### Analysis of nucleus basalis of Meynert volumes

**Fig. 1:**
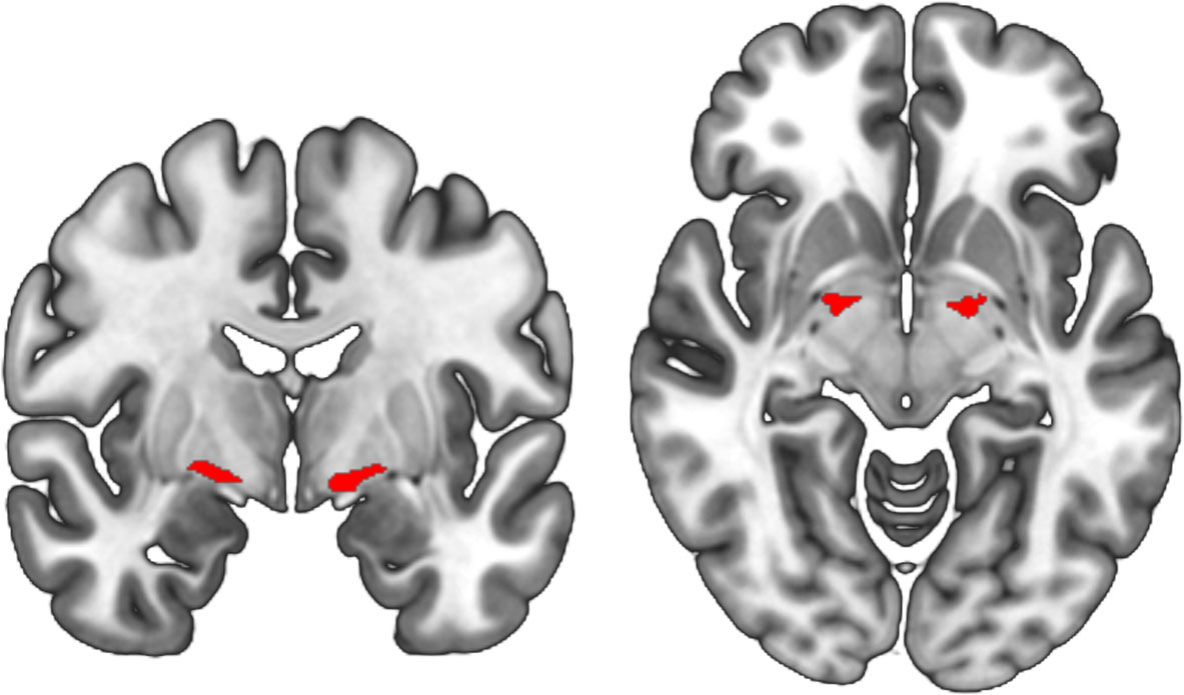
Nucleus basalis of Meynert mask. Region of interest mask for the NBM in MNI space, estimated from the SPM Anatomy Toolbox

The NBM was identified using a probabilistic anatomical map from the SPM Anatomy Toolbox [29] which was created using microscopic delineations of ten post-mortem human brains [30]. The NBM forms part of the basal forebrain which consists of cholinergic cells that can be histologically defined as Ch1–Ch6 where Ch4 corresponds to the NBM [31]. A region of interest mask for the NBM was created using the SPM Anatomy Toolbox (see Fig. 1). NBM volume was averaged across the right and left hemispheres. For each participant, grey matter volume within the mask was calculated. Additionally, the total intracranial volume was calculated in SPM to normalise NBM volume.

### Statistics

Individual alpha peak frequency, alpha reactivity, and alpha power (eyes open and eyes closed) were compared between the groups using univariate ANOVAs or Kruskal-Wallis ANOVAs depending on whether the data were normally distributed. Post hoc tests were Bonferroni-corrected for multiple comparisons. The same analysis was used to compare NBM volume (corrected for total intracranial volume) between groups. Correlations between alpha reactivity and NBM volume were computed using Spearman’s correlations, across all groups and separately in each clinical group. Additionally, Spearman’s correlations between alpha reactivity and NBM volumes and clinical scores were tested for the Mayo Fluctuation Scale (total score and cognitive subscore) and for the MMSE as a measure of overall cognition, in each dementia group separately. False discovery rate (FDR) correction was used to correct correlation *p* values for multiple comparisons.

To assess the influence of dopaminergic medication, the EEG measures were compared between those LBD patients taking dopaminergic medication (*N* = 28) and those not taking dopaminergic medication (*N* = 13) using two-sample *t* tests. Additionally, we assessed Spearman’s correlations between levodopa equivalent daily dose (LEDD) [32] and the EEG measures in those LBD patients who were on dopaminergic medication.

## Results

### Demographics

All three groups were similar in age (see Table 1). Although not statistically significant, there was a tendency for group differences in terms of gender, i.e. LBD patients were predominantly male whereas gender was more balanced in the AD group. To make sure that results were not influenced by these gender differences between groups, all group comparisons were repeated including gender as a covariate. The AD and LBD groups did not differ significantly with respect to overall cognition (MMSE) and dementia duration. As expected, the LBD patients were more impaired than AD in terms of the core LBD symptoms of Parkinsonism, cognitive fluctuations, and visual hallucinations. The percentage of patients taking cholinesterase inhibitors was similar in both dementia groups whereas the majority of LBD patients were taking dopaminergic medication compared to none of the AD patients.

**Table 1:** Demographic and clinical variables, mean (standard deviation)

	HC (*N* = 40)	AD (*N* = 21)	LBD (*N* = 41)	Group differences
Male to female	25:15	14:7	35:6	*χ*^2^ = 5.8, *p* = 0.06^a^
Age	73.4 (6.6)	74.7 (7.2)	74.6 (6.5)	*F*(2, 99) = 0.4, *p* = 0.66^b^
AChEI	–	20	35	*χ*^2^ = 1.4, *p* = 0.25^c^
PD meds	–	0	28	*χ*^2^ = 26.2, *p* < 0.001^c^
Duration	–	4.1 (2.4)^f^	3.2 (2.1)^g^	*U* = 270, *p* = 0.07^d^
MMSE	28.8 (1.1)	21.6 (3.7)	23.1 (3.8)	*t*_60_ = 1.5, *p* = 0.15^e^
UPDRS III	3.9 (4.2)	1.7 (1.5)	20.2 (8.6)	*t*_60_ = 9.7, *p* < 0.001^e^
CAF total	–	0.3 (0.7)^h^	5.2 (4.3)^j^	*t*_56_ = 5.1, *p* < 0.001^e^
Mayo total	–	9.4 (4.4)^h^	14.3 (5.5)^j^	*t*_56_ = 3.5, *p* = 0.001^e^
Mayo cogn	–	2.0 (1.9)^h^	3.0 (1.8)^j^	*t*_56_ = 2.0, *p* = 0.05^e^
NPI total	–	7.4 (7.2)^h^	14.5 (10.5)^g^	*t*_58_ = 2.7, *p* = 0.009^e^
NPI hall	–	0.05 (0.2)^h^	2.0 (2.0)^g^	*t*_58_ = 4.5, *p* < 0.001^e^

*AChEI* number of patients taking acetylcholinesterase inhibitors, *AD* Alzheimer’s disease, *CAF total* Clinician Assessment of Fluctuation total score, *Duration* duration of cognitive symptoms in years, *HC* healthy controls, *LBD* Lewy body dementia, *Mayo total* Mayo Fluctuation Scale, *Mayo cognitive* Mayo Fluctuation cognitive subscale, *MMSE* Mini-Mental State Examination, *PD meds* number of patients taking dopaminergic medication for the management of Parkinson’s disease symptoms, *UPDRS III* Unified Parkinson’s Disease Rating Scale III (motor subsection), *NPI* Neuropsychiatric Inventory, *NPI hall* NPI hallucination subscore

^a^Chi-square test HC, AD, LBD

^b^One-way ANOVA HC, AD, LBD

^c^Chi-square test AD, LBD

^d^Mann-Whitney *U* test AD, LBD

^e^Student’s *t* test AD, LBD

^f^*N* = 19

^g^*N* = 40

^h^*N* = 20

^j^*N* = 38

When considering only the participants that were included in the combined EEG-MRI analysis, all groups were still matched for age and gender, and the dementia groups were matched for overall cognition (Supplementary Table S1).

The DLB and PDD subgroups were comparable in age, gender, overall cognition, dementia duration, the percentage of patients taking cholinesterase inhibitors, cognitive fluctuations, and visual hallucinations (Supplementary Table S2). More PDD patients were taking dopaminergic medication compared to DLB, the levodopa equivalent daily dose (LEDD) was higher in PDD compared to DLB, and they had worse Parkinsonism and total NPI symptom scores.

### Alpha reactivity

Individual alpha peak frequency was significantly lower in both dementia groups compared to controls with no significant difference between LBD and AD (Table 2).

**Table 2:** Group comparison of EEG characteristics and NBM volume

	HC	AD	LBD	Group comparison
Individual alpha peak	8.8 [8.4, 9.2]	7.4 [6.3, 8.4]	6.4 [6.1, 6.7]	*F*_2_ = 42.1, *p* < 0.001^a^
				*p* (HC, AD) = 0.004
				*p* (HC, LBD) < 0.001
				*p* (AD, LBD) = 0.092
Alpha reactivity	0.56 [0.50, 0.63]	0.24 [0.12, 0.34]	0.08 [0.03, 0.14]	*F*_2_ = 59.9, *p* < 0.001^b^
				*p* (HC, AD) < 0.001
				*p* (HC, LBD) < 0.001
				*p* (AD, LBD) = 0.014
Eyes-closed alpha power	49.1 [41.9, 56.3]	34.4 [27.5, 41.3]	39.7 [35.8, 43.5]	*F*_2_ = 7.9, *p* = 0.019^a^
				*p* (HC, AD) = 0.018
				*p* (HC, LBD) = 0.25
				*p* (AD, LBD) = 0.56
Eyes-open alpha power	18.9 [16.1, 21.7]	24.2 [19.4, 29.0]	36.7 [32.4, 41.1]	*F*_2_ = 35.5, *p* < 0.001^a^
				*p* (HC, AD) = 0.25
				*p* (HC, LBD) < 0.001
				*p* (AD, LBD) = 0.005
NBM volume	0.19 [0.18, 0.20]	0.17 [0.16, 0.17]	0.16 [0.15, 0.17]	*F*_2_ = 13.3, *p* < 0.001^b^
				*p* (HC, AD) = 0.003
				*p* (HC, LBD) < 0.001
				*p* (AD, LBD) = 1.0

Mean [95% confidence interval]. Alpha power and alpha reactivity estimated from electrodes O1, Oz, and O2 using individual alpha peak frequencies. NBM volume normalised to total intracranial volume. Group differences assessed by univariate ANOVA or Kruskal-Wallis ANOVA with post hoc tests corrected for multiple comparisons

*AD* Alzheimer’s disease, *HC* healthy controls, *LBD* Lewy body dementia, *NBM* nucleus basalis of Meynert

^a^Kruskal-Wallis ANOVA

^b^Univariate ANOVA

**Fig. 2:**
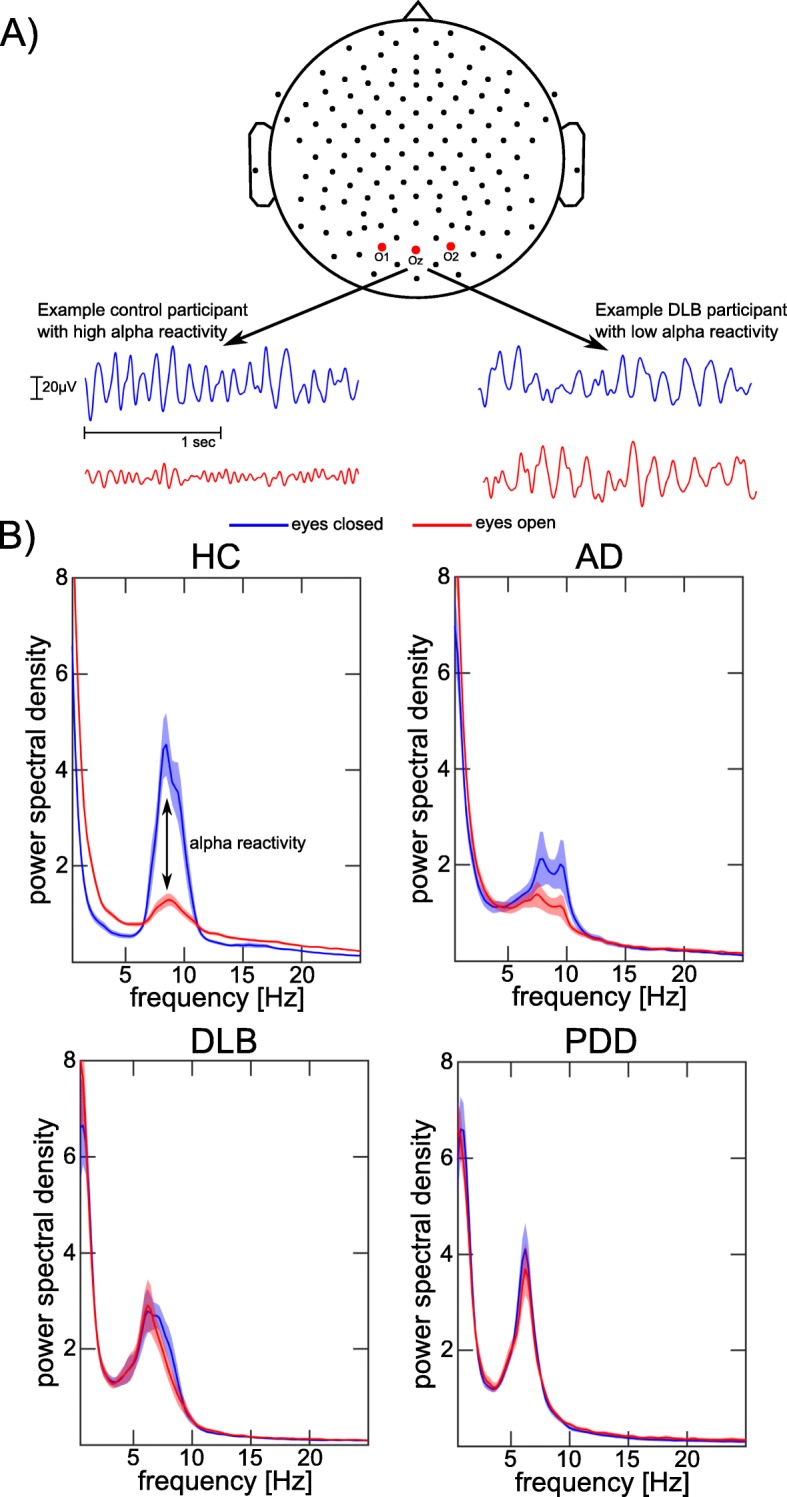
Alpha reactivity analysis. **a** Occipital EEG signals of example control and DLB participants in eyes-closed (blue) and eyes-open (red) conditions. **b** Comparison of mean power spectra for eyes-closed and eyes-open conditions for the different clinical groups. Shaded areas indicate standard errors. AD, Alzheimer’s disease; DLB, dementia with Lewy bodies; HC, healthy controls; PDD, Parkinson’s disease dementia

**Fig. 3:**
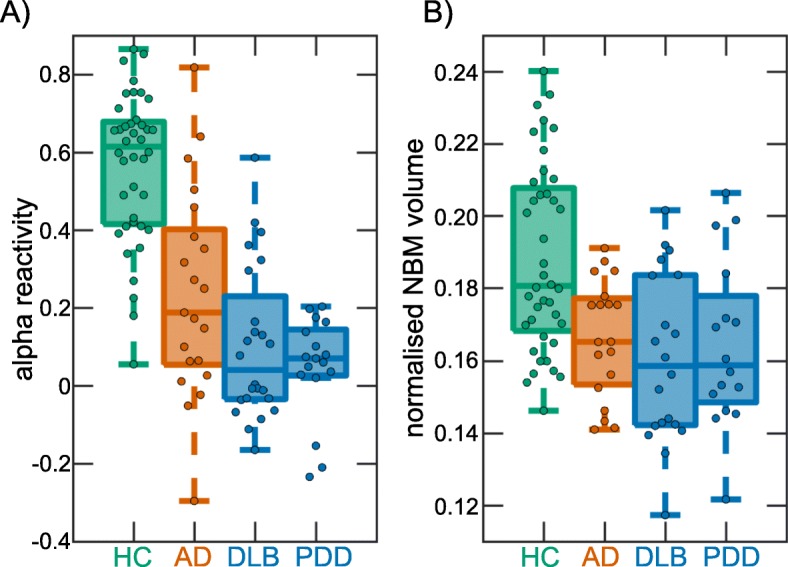
Group comparison. **a** Group comparison of alpha reactivity. **b** Group comparison of NBM volumes (normalised with respect to total intracranial volume). In each boxplot, the central line corresponds to the sample median; the upper and lower borders of the box represent the 25th and 75th percentile, respectively; and the length of the whiskers is 1.5 times the interquartile range. Corresponding results from statistical comparisons between the groups are presented in Table 2. AD, Alzheimer’s disease; DLB, dementia with Lewy bodies; HC, healthy controls; NBM, nucleus basalis of Meynert; PDD, Parkinson’s disease dementia

Alpha reactivity was reduced in both dementia groups compared to controls and was significantly more reduced in LBD compared to AD (Table 2 and Fig. 3a). Eyes-closed alpha power was reduced in AD compared to controls, but there were no significant differences between LBD and controls or between AD and LBD. In contrast, eyes-open alpha power (taking into account individual alpha peak frequencies) was significantly increased in LBD compared to both controls and AD while there was no significant difference between AD and controls (Fig. 2b).

There were no significant differences between the DLB and PDD subgroups in terms of individual alpha peak frequency, alpha reactivity, or eyes-closed and eyes-open alpha power (see Supplementary Table S3).

There was a significant positive correlation between individual alpha peak frequency and alpha reactivity in the AD group (*ρ* = 0.77, *p* < 0.001), but no significant correlations in DLB (*ρ* = 0.37, *p* = 0.08), PDD (*ρ* = 0.33, *p* = 0.19), or controls (*ρ* = 0.12, *p* = 0.46).

The results with respect to alpha reactivity did not change when considering the standard alpha frequency band from 8 to 12 Hz instead of individual alpha peak frequencies (see Section 3 of the Supplementary Material).

Results from all group comparisons did not change when including gender as a covariate (see Supplementary Table S5).

### NBM volume

Normalised NBM volume was decreased in the AD and LBD groups compared to controls; however, there was no significant difference between the dementia groups (see Table 2 and Fig. 3b). There was no difference in normalised NBM volume between the DLB and PDD subgroups (see Supplementary Table S3).

Mean (standard deviation) of total intracranial volumes in litres were 1.44 (0.11) in the control group, 1.42 (0.13) in the AD group, and 1.54 (0.15) in the LBD group.

### Association between alpha reactivity and NBM volume

**Fig. 4:**
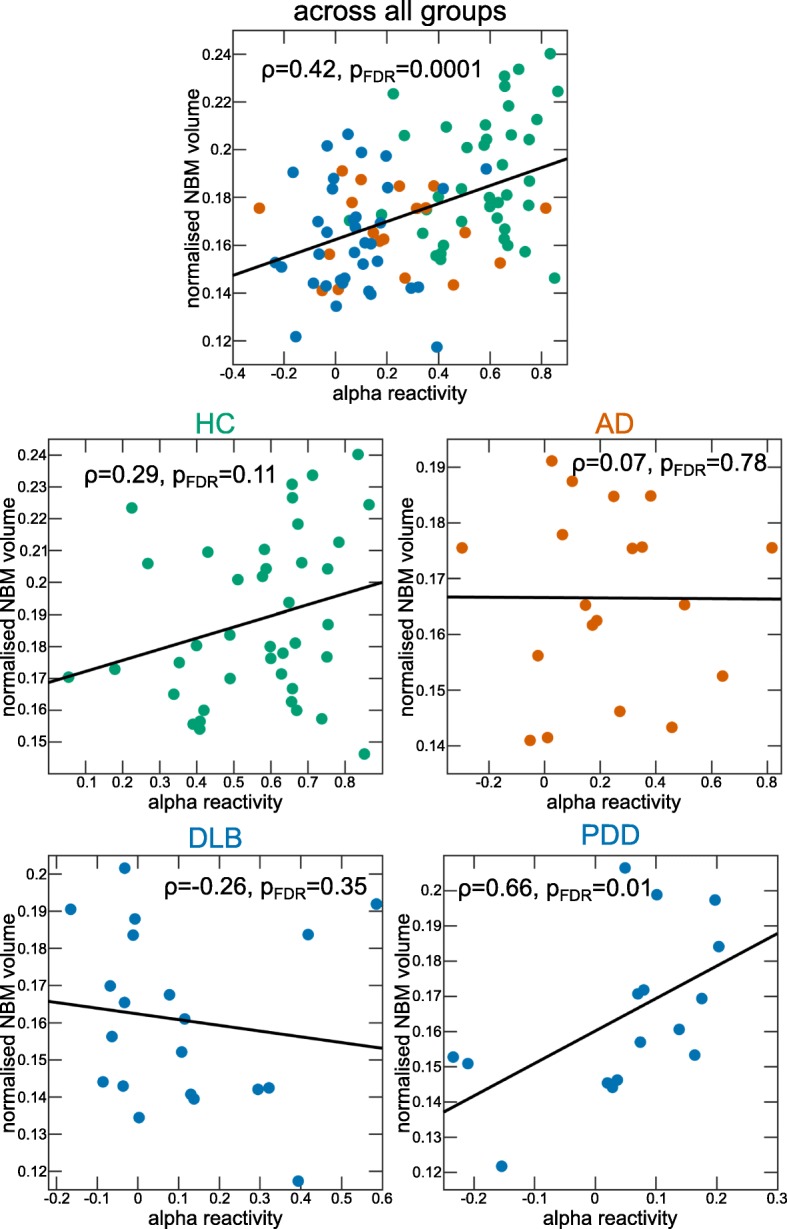
Correlations between alpha reactivity and NBM volume. Spearman’s correlations between alpha reactivity and normalised NBM volume across all groups and in each group separately. *p* values are FDR (false discovery rate)-corrected for multiple comparisons. AD, Alzheimer’s disease; DLB, dementia with Lewy bodies; FDR, false discovery rate; HC, healthy controls; NBM, nucleus basalis of Meynert; PDD, Parkinson’s disease dementia

When considering the whole group (across AD, LBD, and controls), there was a significant positive correlation between alpha reactivity and NBM volume (*ρ* = 0.42, *p*_FDR_ = 0.0001, Fig. 4). When considering each group separately, there was a significant positive correlation in the PDD group (*ρ* = 0.66, *p*_FDR_ = 0.01), whereas there were no significant correlations in the other three groups (all *p* > 0.1).

Correlations between alpha reactivity and NBM volume were similar when using the standard alpha frequency band (Section 3 of the Supplementary Material).

### Correlations with clinical scores

In PDD, alpha reactivity was positively correlated with MMSE (*ρ* = 0.51, *p* = 0.035). However, this correlation did not survive correction for multiple comparisons. All other correlations between alpha reactivity and NBM volumes and clinical scores were not significant (all *p* > 0.1).

### Effect of dopaminergic medication in the LBD group

There were no significant differences between LBD patients who were taking dopaminergic medication compared to those not taking these medications (Supplementary Table S6). Furthermore, in those patients who were taking dopaminergic medication, there were no significant correlations between any EEG measures and LEDD (Supplementary Table S6).

## Discussion

In this study, we investigated EEG alpha reactivity in patients with LBD compared to AD and healthy controls and its relation to cholinergic system integrity as measured by NBM volume. We found a reduction in alpha reactivity in the dementia groups compared to controls which is in line with previous EEG studies in AD [19, 20] and MEG studies in DLB [21] and PDD [22]. Importantly, for the first time, we also showed that alpha reactivity is more severely affected in DLB and PDD compared to AD. Furthermore, in agreement with previous findings in healthy participants [17, 18], we found evidence for an involvement of the cholinergic system in modulating alpha reactivity, specifically in PDD patients.

**Fig. 5:**
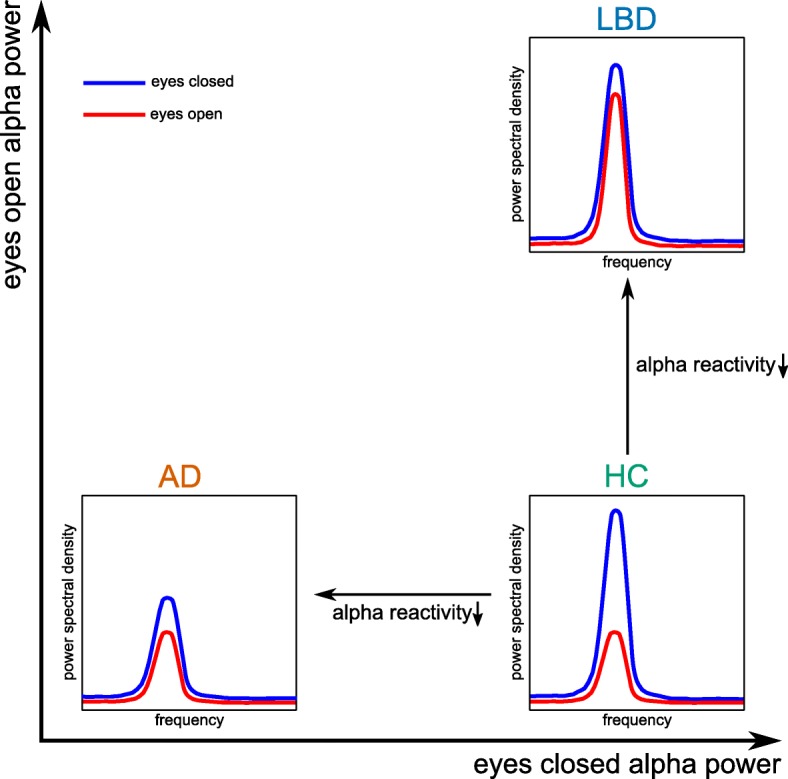
Interpretation of alpha reactivity changes. Illustration of how a reduction in alpha reactivity can be mainly due to a decrease in eyes-closed alpha power (in AD) or an increase in eyes-open alpha power (in LBD) compared to controls. AD, Alzheimer’s disease; LBD, Lewy body dementia; HC, healthy controls

Since alpha reactivity is determined by the difference between eyes-closed and eyes-open alpha power, a reduction in alpha reactivity as observed in the dementia groups can occur in two different ways (see Fig. 5). In AD, eyes-closed alpha power was reduced compared to controls while eyes-open alpha power was not significantly different from healthy control levels. In the LBD group, the opposite was the case: while eyes-closed alpha power was not significantly reduced compared to controls, eyes-open alpha power was significantly increased compared to controls and AD (after taking into account individual alpha peak frequencies). Furthermore, in AD, there was a strong positive correlation between alpha reactivity and individual alpha peak frequency, indicating that the reduction in alpha reactivity in this group might be more related to general alpha power reduction and alpha slowing [26]. In contrast, in LBD, even though the general EEG slowing was slightly more severe than in AD, there was no significant correlation between alpha reactivity and alpha slowing, indicating that the loss of alpha reactivity might be a process that is more independent from general EEG slowing than in AD. Instead, loss of alpha reactivity in LBD might be more related to a lack of neuronal desynchronization upon opening the eyes.

In the healthy human brain, opening of the eyes normally leads to a suppression of alpha power due to neuronal desynchronization [15, 16]. This state of low alpha power has been associated with highest levels of cortical responsiveness and has been suggested to be a more externally oriented brain state in which it is easiest for external stimuli to reach the cortex [33]. In contrast, higher eyes-open alpha power indicates a more internally oriented state making it harder for external stimuli to be perceived [33]. Evidence comes from studies of pre-stimulus alpha power which showed that performance is highest if pre-stimulus alpha power is low [34]. However, the extent of alpha power suppression is not only important for processing of visual stimuli, but has also been related to attention and cognitive performance more generally. Low alpha power has been associated with higher activity in attention networks [35]. Furthermore, the extent of alpha suppression upon eyes opening has been shown to be positively correlated with cognitive performance [36] and has been related to attention and cognitive load [37]. Simultaneous EEG-fMRI studies have reported a negative relationship between blood oxygen level dependent (BOLD) signal and alpha power indicating a simultaneous occurrence of occipital alpha power decrease and neuronal activation in the occipital cortex and other cortical areas [38, 39].

The increase in eyes-open alpha power in LBD might therefore indicate a specific impairment in neuronal desynchronization. Instead of activating neurons in primary and secondary visual areas when opening the eyes, the cortex of LBD patients seems to stay in a more synchronised state which might lead to a loss of cortical responsiveness. This in turn might lead to problems with attention and cognition [36, 37].

Several previous studies have investigated the mechanisms that lead to neuronal desynchronization when opening the eyes and thereby modulate alpha reactivity. There is compelling evidence for a role of the cholinergic system [17, 18]. The suppression of alpha power from eyes closed to eyes open has been shown to be related to an increase in functional connectivity between the NBM and primary visual areas [17]. Furthermore, white matter integrity along fibre tracts connecting the NBM with occipital areas was negatively correlated with alpha reactivity. These findings suggest that cholinergic drive from the NBM might play an important role in modulating the reduction in alpha power from eyes closed to eyes open [17]. Additionally, Osipova et al. [18] showed that alpha power suppression upon opening the eyes was impaired when cholinergic neurotransmission was temporarily blocked by the cholinergic antagonist scopolamine, further suggesting that the integrity of the cholinergic system is crucial for alpha power suppression.

In the present study, across all groups and in the PDD group in particular, loss of alpha reactivity was related to volume loss within the NBM. The failure to activate neural sources in occipital cortex upon opening the eyes might therefore be due to a loss of cholinergic drive from the NBM which is in line with these previous studies [17, 18]. The lack of a specific association between alpha reactivity and NBM volume in DLB might be due to the fact that functional impairment within the cholinergic system can precede structural abnormalities; in particular, it has been shown that even prior to neurodegeneration, alpha-synuclein can reduce cholinergic neurotransmitter production [9]. In contrast, PDD patients with a comparable level of cognitive impairment typically have a longer disease duration which might lead to structural abnormalities within the cholinergic system playing a greater role in the loss of alpha reactivity in these patients. Furthermore, PDD patients show less AD co-pathology than patients with DLB [40], making PDD a “purer” alpha-synucleinopathy which might explain the discrepant findings in these two groups.

The observed association between alpha reactivity and the cholinergic system—if replicated in other studies—might suggest alpha reactivity as an interesting potential measure of treatment response in clinical trials which seek to remediate cholinergic function.

Contrary to our hypothesis, alpha reactivity was not significantly correlated with measures of cognitive fluctuation severity in LBD. This might be due to the fact that alpha reactivity was quite severely reduced in most patients and most of them had cognitive fluctuations, which might have led to a floor effect. The fact that AD patients, who have less severe cognitive fluctuations compared to LBD [25], showed a less severe reduction in alpha reactivity might indicate that loss of alpha reactivity is related to the presence of cognitive fluctuations, while a relationship between alpha reactivity and cognitive fluctuation severity is more difficult to establish based on the present results.

We decided to use individual alpha peak frequencies to determine alpha power instead of using a fixed alpha frequency band (usually from 8 to 12 Hz) [21, 22]. This was done to account for a shift of the alpha peak to slower frequencies in AD and LBD [26, 27]. The mean alpha peak in the LBD group therefore lies within a frequency range that has been termed pre-alpha [41, 42], but is also considered to represent the fast-theta band in other studies [27]. However, we showed that alpha reactivity differences between groups and the association with NBM volume remained the same when repeating the analysis using the standard alpha frequency band.

A potential limitation of the present study is the fact that most dementia patients were taking cholinesterase inhibitors which have been shown to influence the cortical EEG signal by increasing eyes-closed alpha power and reducing slow-wave activity [43–45]. Due to the low number of patients not taking these medications, it was not possible to study the effect of cholinesterase inhibitors on the present results nor would it be ethical to withdraw these medications. However, investigating the effect of cholinergic medication on alpha reactivity in AD and LBD will be an important step of future research which will also help to better understand the relationship between alpha reactivity and cholinergic neurotransmission and evaluate the potential use of alpha reactivity as a predictor of treatment response.

A further potential limitation is the use of dopaminergic medication in many LBD patients which has also been shown to influence EEG signals by increasing eyes-closed alpha power [46, 47]. However, we did not find differences between LBD patients who were taking dopaminergic medication compared to those patients not taking these medications, and there were no significant correlations between LEDD and the EEG measures included in the present study.

## Conclusions

In conclusion, we showed that LBD patients show an impairment in neuronal desynchronization upon opening the eyes which distinguishes these patients from healthy controls and patients with AD and which might be related to a loss of cholinergic drive from the NBM in PDD. While the importance of a general slowing of the EEG signal in LBD has been discussed in many studies [26, 27], changes in eyes-open resting EEG in these patients have not been investigated in detail. The present study therefore complements previous research by showing that studying eyes-open EEG data can provide important new insights into changes in brain state in LBD.

## Supplementary information

**Additional file 1.**

## Data Availability

The data that support the findings of this study are available from the corresponding author, upon reasonable request.

## Abbreviations

AD: Alzheimer’s disease
DLB: Dementia with Lewy bodies
EEG: Electroencephalography
FDR: False discovery rate
LBD: Lewy body dementia
MEG: Magnetencephalography
MMSE: Mini-Mental State Examination
NBM: Nucleus basalis of Meynert
PDD: Parkinson’s disease dementia

## References

[1] McKeith IG, O’Brien JT, Walker Z, Tatsch K, Booij J, Darcourt J (2007). Sensitivity and specificity of dopamine transporter imaging with 123I-FP-CIT SPECT in dementia with Lewy bodies: a phase III, multicentre study. Lancet Neurol.

[2] McKeith IG, Boeve BF, Dickson DW, Halliday G, Aarsland D, Attems J (2017). Diagnosis and management of dementia with Lewy bodies fourth consensus report of the DLB consortium. Neurology.

[3] Tiraboschi P, Hansen LA, Alford M, Merdes A, Masliah E, Thal LJ (2002). Early and widespread cholinergic losses differentiate dementia with Lewy bodies from Alzheimer disease. Arch Gen Psychiatry.

[4] Francis PT, Perry EK (2007). Cholinergic and other neurotransmitter mechanisms in Parkinson’s disease, Parkinson’s disease dementia, and dementia with Lewy bodies. Mov Disord.

[5] Perry EK, Irving D, Kerwin JM, McKeith IG, Thompson P, Collerton D (1993). Cholinergic transmitter and neurotrophic activities in Lewy body dementia. Alzheimer Dis Assoc Disord.

[6] Mesulam M-M (2013). Cholinergic circuitry of the human nucleus basalis and its fate in Alzheimer’s disease. J Comp Neurol.

[7] Colloby SJ, Elder GJ, Rabee R, O’Brien JT, Taylor J-P (2017). Structural grey matter changes in the substantia innominata in Alzheimer’s disease and dementia with Lewy bodies: a DARTEL-VBM study. Int J Geriatr Psychiatry.

[8] Kim HJ, Lee JE, Shin SJ, Sohn YH, Lee PH (2011). Analysis of the substantia innominata volume in patients with Parkinson’s disease with dementia, dementia with Lewy bodies, and Alzheimer’s disease. J Mov Disord.

[9] Hall H, Reyes S, Landeck N, Bye C, Leanza G, Double K (2014). Hippocampal Lewy pathology and cholinergic dysfunction are associated with dementia in Parkinson’s disease. Brain.

[10] Kehagia AA, Barker RA, Robbins TW (2013). Cognitive impairment in Parkinson’s disease: the dual syndrome hypothesis. Neurodegener Dis.

[11] Schulz J, Pagano G, Fernández Bonfante JA, Wilson H, Politis M (2018). Nucleus basalis of Meynert degeneration precedes and predicts cognitive impairment in Parkinson’s disease. Brain.

[12] Hanyu H, Asano T, Sakurai H, Tanaka Y, Takasaki M, Abe K (2002). MR analysis of the substantia innominata in normal aging, Alzheimer disease, and other types of dementia. Am J Neuroradiol.

[13] O’Dowd S, Schumacher J, Burn DJ, Bonanni L, Onofrj M, Thomas A, et al. Fluctuating cognition in the Lewy body dementias. Brain. 2019; Available from: https://academic.oup.com/brain/advance-article/doi/10.1093/brain/awz235/5549757. 10.1093/brain/awz235 31411317

[14] Edwards K, Royall D, Hershey L, Lichter D, Hake A, Farlow M (2007). Efficacy and safety of galantamine in patients with dementia with Lewy bodies: a 24-week open-label study. Dement Geriatr Cogn Disord.

[15] Markand ON. Alpha Rhythms. J Clin Neurophysiol. 1990;7(2):163–90. 10.1097/00004691-199004000-00003 2187019

[16] Könönen M, Partanen JV (1993). Blocking of EEG alpha activity during visual performance in healthy adults. A quantitative study. Electroencephalogr Clin Neurophysiol.

[17] Wan L, Huang H, Schwab N, Tanner J, Rajan A, Lam NB (2019). From eyes-closed to eyes-open: role of cholinergic projections in EC-to-EO alpha reactivity revealed by combining EEG and MRI. Hum Brain Mapp.

[18] Osipova D, Ahveninen J, Kaakkola S, Jääskeläinen IP, Huttunen J, Pekkonen E (2003). Effects of scopolamine on MEG spectral power and coherence in elderly subjects. Clin Neurophysiol.

[19] Fonseca LC, Tedrus GMAS, Fondello MA, Reis IN, Fontoura DS (2011). EEG theta and alpha reactivity on opening the eyes in the diagnosis of Alzheimer’s disease. Clin EEG Neurosci.

[20] Babiloni C, Lizio R, Vecchio F, Frisoni GB, Pievani M, Geroldi C (2011). Reactivity of cortical alpha rhythms to eye opening in mild cognitive impairment and Alzheimer’s disease: an EEG study. J Alzheimer’s Dis.

[21] Franciotti R, Iacono D, Della PS, Pizzella V, Torquati K, Onofrj M (2006). Cortical rhythms reactivity in AD, LBD and normal subjects: a quantitative MEG study. Neurobiol Aging.

[22] Bosboom JLW, Stoffers D, Stam CJ, van Dijk BW, Verbunt J, Berendse HW (2006). Resting state oscillatory brain dynamics in Parkinson’s disease: an MEG study. Clin Neurophysiol.

[23] Emre M, Aarsland D, Brown R, Burn DJ, Duyckaerts C, Mizuno Y (2007). Clinical diagnostic criteria for dementia associated with Parkinson’s disease. Mov Disord.

[24] McKhann GM, Knopman DS, Chertkow H, Hyman BT, Jack CR, Kawas CH (2011). The diagnosis of dementia due to Alzheimer’s disease: recommendations from the National Institute on Aging-Alzheimer’s Association workgroups on diagnostic guidelines for Alzheimer’s disease. Alzheimers Dement.

[25] Ferman TJ, Smith GE, Boeve BF, Ivnik RJ, Petersen RC, Knopman D (2004). DLB fluctuations: specific features that reliably differentiate DLB from AD and normal aging. Neurol Int.

[26] Peraza LR, Cromarty RA, Kobeleva X, Firbank MJ, Killen A, Graziadio S (2018). Electroencephalographic derived network differences in Lewy body dementia compared to Alzheimer’s disease patients. Sci Rep.

[27] Stylianou M, Murphy N, Peraza LR, Graziadio S, Cromarty RA, Killen A (2018). Quantitative electroencephalography as a marker of cognitive fluctuations in dementia with Lewy bodies and an aid to differential diagnosis. Clin Neurophysiol.

[28] Ashburner J (2007). A fast diffeomorphic image registration algorithm. Neuroimage.

[29] Eickhoff SB, Stephan KE, Mohlberg H, Grefkes C, Fink GR, Amunts K (2005). A new SPM toolbox for combining probabilistic cytoarchitectonic maps and functional imaging data. Neuroimage.

[30] Zaborszky L, Hoemke L, Mohlberg H, Schleicher A, Amunts K, Zilles K (2008). Stereotaxic probabilistic maps of the magnocellular cell groups in human basal forebrain. Neuroimage.

[31] Mesulam M-M, Mufson EJ, Levey AI, Wainer BH (1983). Cholinergic innervation of cortex by the basal forebrain: cytochemistry and cortical connections of the septal area, diagonal band nuclei, nucleus basalis (substantia innominata), and hypothalamus in the rhesus monkey. J Comp Neurol.

[32] Tomlinson CL, Stowe R, Patel S, Rick C, Gray R, Clarke CE (2010). Systematic review of levodopa dose equivalency reporting in Parkinson’s disease. Mov Disord.

[33] Hanslmayr S, Gross J, Klimesch W, Shapiro KL (2011). The role of alpha oscillations in temporal attention. Brain Res Rev.

[34] Hanslmayr S, Aslan A, Staudigl T, Klimesch W, Herrmann CS, Bäuml K-H (2007). Prestimulus oscillations predict visual perception performance between and within subjects. Neuroimage.

[35] Sadaghiani S, Scheeringa R, Lehongre K, Morillon B, Giraud A-L, Kleinschmidt A (2010). Intrinsic connectivity networks, alpha oscillations, and tonic alertness: a simultaneous electroencephalography/functional magnetic resonance imaging study. J Neurosci.

[36] Klimesch W (1999). EEG alpha and theta oscillations reflect cognitive and memory performance: a review and analysis. Brain Res Rev.

[37] Stipacek A, Grabner RH, Neuper C, Fink A, Neubauer AC (2003). Sensitivity of human EEG alpha band desynchronization to different working memory components and increasing levels of memory load. Neurosci Lett.

[38] Feige B, Scheffler K, Esposito F, Di Salle F, Hennig J, Seifritz E (2005). Cortical and subcortical correlates of electroencephalographic alpha rhythm modulation. J Neurophysiol.

[39] Moosmann M, Ritter P, Krastel I, Brink A, Thees S, Blankenburg F (2003). Correlates of alpha rhythm in functional magnetic resonance imaging and near infrared spectroscopy. Neuroimage.

[40] Gomperts SN, Rentz DM, Moran E, Becker JA, Locascio JJ, Klunk WE (2008). Imaging amyloid deposition in Lewy body diseases. Neurol Int.

[41] Bonanni L, Thomas A, Tiraboschi P, Perfetti B, Varanese S, Onofrj M (2008). EEG comparisons in early Alzheimer’s disease, dementia with Lewy bodies and Parkinson’s disease with dementia patients with a 2-year follow-up. Brain.

[42] Bonanni L, Franciotti R, Nobili F, Kramberger MG, Taylor JP, Garcia-Ptacek S (2016). EEG markers of dementia with Lewy bodies: a multicenter cohort study. J Alzheimers Dis.

[43] Babiloni C, Del Percio C, Bordet R, Bourriez JL, Bentivoglio M, Payoux P (2013). Effects of acetylcholinesterase inhibitors and memantine on resting-state electroencephalographic rhythms in Alzheimer’s disease patients. Clin Neurophysiol.

[44] Onofrj M, Thomas A, Iacono D, Luciano AL, Di Iorio A (2003). The effects of a cholinesterase inhibitor are prominent in patients with fluctuating cognition: a part 3 study of the main mechanism of cholinesterase inhibitors in dementia. Clin Neuropharmacol.

[45] Fogelson N, Kogan E, Korczyn AD, Giladi N, Shabtai H, Neufeld MY (2003). Effects of rivastigmine on the quantitative EEG in demented Parkinsonian patients. Acta Neurol Scand.

[46] Babiloni C, Del Percio C, Lizio R, Noce G, Lopez S, Soricelli A, et al. Levodopa may affect cortical excitability In Parkinson’s disease patients with cognitive deficits as revealed by reduced activity of cortical sources of resting state electroencephalographic rhythms. Neurobiol Aging. 2018; Available from: https://linkinghub.elsevier.com/retrieve/pii/S0197458018302938. Elsevier Inc. 10.1016/j.neurobiolaging.2018.08.010 30312790

[47] Melgari J-M (2014). Alpha and beta EEG power reflects L-dopa acute administration in parkinsonian patients. Front Aging.

