# Supplementary material for: EEG alpha reactivity and cholinergic system integrity in Lewy body dementia and Alzheimer’s disease
Source: Alzheimers Res Ther. 2020 Apr 22;12:46. doi: 10.1186/s13195-020-00613-6 (PMC7178985; doi:10.1186/s13195-020-00613-6)
Supplement: Supplementary file 1 — Additional file 1. [file 13195_2020_613_MOESM1_ESM.pdf]

Supplementary Material:
EEG alpha reactivity and cholinergic system integrity in Lewy body dementia and Alzheimer's disease

Julia Schumacher, Alan J. Thomas, Luis R. Peraza, Michael Firbank, Ruth Cromarty, Calum Hamilton, Paul C. Donaghy, John T. O'Brien, John-Paul Taylor

Correspondence: julia.schumacher@newcastle.ac.uk

Content

1. Demographics for EEG-MRI group
2. Comparison of DLB and PDD subgroups
3. Alpha reactivity analysis using the standard alpha frequency band
4. Group comparisons including gender as covariate
5. Effect of dopaminergic medication in the LBD group

1. Demographics for EEG-MRI group

Table S1. Demographic and clinical variables for participants included in the combined EEG-MRI analysis, mean (standard deviation).

	HC (N=40)	AD (N=19)	LBD (N=36*)	Group differences
Male: female	25:15	13:6	31:5	χ^2=5.5, p=0.06[a]
Age	73.4 (6.6)	75.4 (7.1)	75.0 (6.3)	F(2,92)=0.8, p=0.45[b]
AChEI	-	19	32	χ^2=2.3, p=0.13[c]
PD meds	-	0	25	χ^2=24.2, p<0.001[c]
Duration	-	3.7 (1.7)[f]	3.3 (2.2)[g]	U=246, p=0.18[d]
MMSE	28.8 (1.1)	22.0 (3.4)	23.3 (3.9)	t_{53}=1.2, p=0.24[e]
UPDRS III	3.9 (4.2)	1.7 (1.4)	20.4 (8.5)	t_{53}=9.5, p<0.001[e]
CAF total	-	0.3 (0.8)[f]	5.0 (4.3)[h]	t_{50}=4.6, p<0.001[e]
Mayo total	-	9.1 (4.2)[f]	14.2 (5.7)[h]	t_{50}=3.4, p=0.001[e]
Mayo cogn	-	2.0 (2.0)[f]	2.9 (1.8)[h]	t_{50}=1.7, p=0.10[e]
NPI total	-	7.3 (7.3)[f]	14.8 (11.1)[j]	t_{52}=2.6, p=0.012[e]
NPI hall	-	0.06 (0.2)[f]	2.0 (2.0)[j]	t_{52}=4.1, p<0.001[e]

AChEI, number of patients taking acetylcholinesterase inhibitors; AD, Alzheimer's disease; CAF total, Clinician Assessment of Fluctuation total score; Duration, duration of cognitive symptoms in years; HC, healthy controls; LBD, Lewy body dementia; Mayo total, Mayo Fluctuations Scale; Mayo cognitive, Mayo Fluctuation cognitive subscale; MMSE, Mini Mental State Examination; PD meds, number of patients taking dopaminergic medication for the management of Parkinson's disease symptoms; UPDRS III, Unified Parkinson's Disease Rating Scale III (motor subsection); NPI, Neuropsychiatric Inventory; NPI hall, NPI hallucination subscore

[a] Chi-square test HC, AD, LBD; [b] One-way ANOVA HC, AD, LBD; [c] Chi-square test AD, LBD; [d] Mann Whitney U test AD, LBD; [e] Student's t-test AD, LBD.

[f] N=18, [g] N=35, [h] N=34, [j] N=36

* 20 DLB and 16 PDD

2. Comparison of DLB and PDD subgroups

Table S2. Demographic and clinical variables comparing DLB and PDD subgroups, mean (standard deviation).

	DLB (N=24)	PDD (N=17)	Group differences
Male: female	19:5	16:1	χ^2=1.8, p=0.18[a]
Age	76.2 (6.4)	72.4 (6.1)	t_{39}=1.9, p=0.06[b]
AChEI	22	13	χ^2=1.8, p=0.18[a]
PD meds	11	17	χ^2=13.5, p<0.001[a]
LEDD	374.7 (205.4)	867.5 (365.5)	t_{26}=4.1, p<0.001[b]
Duration	3.5 (2.4)	2.7 (1.5)[d]	U=159, p=0.36[c]
MMSE	22.6 (4.4)	23.8 (2.7)	t_{39}=1.0, p=0.31[b]
UPDRS III	16.2 (7.7)	25.8 (6.7)	t_{39}=4.1, p<0.001[b]
CAF total	4.3 (4.1)[e]	6.7 (4.3)[f]	t_{39}=1.7, p=0.10[b]
Mayo total	13.4 (6.0)[e]	15.7 (4.5)[f]	t_{39}=1.3, p=0.21[b]
Mayo cogn	2.7 (1.7)[e]	3.4 (1.7)[f]	t_{39}=1.3, p=0.20[b]
NPI total	10.2 (6.4)[e]	20.3 (12.3)	t_{39}=3.4, p=0.002[b]
NPI hall	1.8 (1.9)[e]	2.4 (2.1)	t_{39}=0.9, p=0.37[b]

AChEI, number of patients taking acetylcholinesterase inhibitors; CAF total, Clinician Assessment of Fluctuation total score; DLB, Dementia with Lewy bodies; Duration, duration of cognitive symptoms in years; LEDD, levodopa equivalent daily dose; Mayo total, Mayo Fluctuations Scale; Mayo cognitive, Mayo Fluctuation cognitive subscale; MMSE, Mini Mental State Examination; PDD, Parkinson's disease dementia; PD meds, number of patients taking dopaminergic medication for the management of Parkinson's disease symptoms; UPDRS III, Unified Parkinson's Disease Rating Scale III (motor subsection); NPI, Neuropsychiatric Inventory; NPI hall, NPI hallucination subscore

[a] Chi-square test DLB, PDD; [b] Student's t-test DLB, PDD; [c] Mann Whitney U test DLB, PDD.

[d] N=16, [e] N=23, [f] N=15.

Table S3. Mean [95% confidence interval]. Alpha power and alpha reactivity estimated from electrodes O1, Oz, and O2 using individual alpha peak frequencies. NBM volume normalized to total intracranial volume. Differences between DLB and PDD were assessed by Student's t-tests.

	DLB	PDD	Group comparison
individual alpha peak	6.6 [6.1, 7.0]	6.2 [5.9, 6.5]	t_{39}=1.3, p=0.20
alpha reactivity	0.10 [0.02,0.19]	0.05 [-0.02,0.11]	t_{39}=1.0, p=0.31
eyes closed alpha power	40.3 [34.8, 45.9]	38.7 [33.1, 44.3]	t_{39}=0.42, p=0.68
eyes open alpha power	37.0 [30.1, 43.9]	36.3 [31.5, 41.1]	t_{39}=0.15, p=0.88
NBM volume	0.16 [0.15, 0.17]	0.16 [0.15, 0.18]	t_{39}=0.47, p=0.64

3. Alpha reactivity analysis using the standard alpha frequency band

Instead of using alpha power from individual alpha peak frequencies to estimate alpha reactivity, we performed the same analysis using the standard alpha frequency band from 8-12 Hz (Wan et al., 2019).
Alpha reactivity was reduced in both dementia groups compared to controls with a significantly greater reduction in LBD compared to AD (Supplementary Table S3).
When considering the whole group (across AD, LBD, and controls), there was a significant positive correlation between alpha reactivity and total NBM volume ($\rho=0.42$, $p<0.001$).
When considering each group separately, there was a significant positive correlation in the PDD group ($\rho=0.66$, $p=0.006$), whereas there were no significant correlations in the other three groups (all $p>0.05$).

Table S4. Mean [95% confidence interval]. Alpha power and alpha reactivity estimated from electrodes O1, Oz, and O2 using the standard alpha frequency band from 8-12 Hz. Group differences assessed by Kruskal-Wallis ANOVA with post-hoc tests corrected for multiple comparisons.

	HC	AD	LBD	Group comparison
alpha reactivity	0.50 [0.42, 0.59]	0.25 [0.12, 0.38]	-0.01 [-0.09,0.06]	$F_2=49.3$, $p<0.001$<br>p(HC,AD)=0.01<br>p(HC,LBD)<0.001<br>p(AD,LBD)=0.01

AD, Alzheimer's disease; HC, healthy controls; LBD, Lewy body dementia

4. Group comparisons including gender as covariate

Table S5. Group comparison of EEG characteristics and NBM volume, including gender as covariate.

	HC	AD	LBD	Group comparison
individual alpha peak	8.8 (0.23)	7.4 (0.32)	6.4 (0.23)	F_2=25.3, p<0.001<br>p(HC,AD)= 0.002<br>p(HC,LBD)<0.001<br>p(AD,LBD)=0.06
alpha reactivity	0.56 (0.03)	0.23 (0.04)	0.09 (0.03)	F_2=54.3, p<0.001<br>p(HC,AD)<0.001<br>p(HC,LBD)<0.001<br>p(AD,LBD)=0.032
eyes closed alpha power	47.8 (2.64)	33.6 (3.61)	41.4 (2.62)	F_2=5.1, p=0.008<br>p(HC,AD)= 0.006<br>p(HC,LBD)=0.28<br>p(AD,LBD)=0.26
eyes open alpha power	18.5 (1.81)	24.0 (2.48)	37.2 (1.80)	F_2=26.7, p<0.001<br>p(HC,AD)= 0.23<br>p(HC,LBD)<0.001<br>p(AD,LBD)<0.001
NBM volume	0.19 (0.004)	0.17 (0.005)	0.16 (0.004)	F_2=10.8, p<0.001<br>p(HC,AD)=0.003<br>p(HC,LBD)<0.001<br>p(AD,LBD)=1.0

Mean (standard error), adjusted for gender covariate. Alpha power and alpha reactivity estimated from electrodes O1, Oz, and O2 using individual alpha peak frequencies. NBM volume normalized to total intracranial volume. Group differences assessed by univariate ANCOVA including gender as covariate, with post-hoc tests corrected for multiple comparisons.

AD, Alzheimer's disease; HC, healthy controls; LBD, Lewy body dementia; NBM, nucleus basalis of Meynert

5. Effect of dopaminergic medication in the LBD group

Table S6. Mean (standard deviation). Alpha power and alpha reactivity estimated from electrodes O1, Oz, and O2 using individual alpha peak frequencies. NBM volume normalized to total intracranial volume. Differences between LBD patients on dopaminergic mediation and LBD patients not on dopaminergic medication were assessed by two-sample Student's t-tests.

	LBD on dopaminergic medication (N=28)	LBD not on dopaminergic medication (N=13)	Group comparison	Correlation with LEDD
individual alpha peak	6.4 (0.86)	6.3 (1.11)	t_{39}=0.3, p=0.80	ρ=-0.02, p=0.93
alpha reactivity	0.09 (0.18)	0.05 (0.15)	t_{39}=0.7, p=0.46	ρ=-0.27, p=0.17
eyes closed alpha power	39.1 (10.9)	40.9 (14.8)	t_{39}=0.5, p=0.65	ρ=0.15, p=0.45
eyes open alpha power	35.3 (12.2)	39.8 (16.8)	t_{39}=1.0, p=0.34	ρ=0.27, p=0.16

LBD, Lewy body dementia; LEDD, levodopa equivalent daily dose
